# Regulation of type 1 iodothyronine deiodinase by LXRα

**DOI:** 10.1371/journal.pone.0179213

**Published:** 2017-06-15

**Authors:** Yoriko Sakane, Naotetsu Kanamoto, Ichiro Yamauchi, Tetsuya Tagami, Yusuke Morita, Masako Miura, Masakatsu Sone, Akihiro Yasoda, Takeshi Kimura, Kazuwa Nakao, Nobuya Inagaki

**Affiliations:** 1 Department of Diabetes, Endocrinology and Nutrition, Kyoto University Graduate School of Medicine, Kyoto, Japan; 2 Division of Endocrinology and Metabolism, Clinical Research Institute, National Hospital Organization Kyoto Medical Center, Kyoto, Japan; 3 Department of Cardiovascular Medicine, Kyoto University Graduate School of Medicine, Kyoto, Japan; 4 Medical Innovation Center, Kyoto University Graduate School of Medicine, Kyoto, Japan; Nihon University School of Medicine, JAPAN

## Abstract

The iodothyronine deiodinases are selenoenzymes that regulate the activity of thyroid hormone via specific inner- or outer-ring deiodination. In humans, type 1 deiodinase (D1) is highly expressed in the liver, but the mechanism by which its gene expression is regulated remains to be elucidated. Liver X receptor α (LXRα), a transcription factor of the nuclear receptor superfamily, is highly expressed in the liver, where it functions as a sensor for excess intracellular oxysterols. LXRα interacts with other nuclear receptors on promoters of genes that contain a binding core sequence for nuclear receptors. In addition, it is reported that the promoter of the gene encoding human D1 (*hDIO1*) contains the core sequence for one of nuclear receptors, thyroid hormone receptor (TR). We investigated the involvement of LXRα in the regulation of *hDIO1*, in the liver. We performed *hDIO1* promoter–reporter assays using a synthetic LXR agonist, T0901317, and compared promoter activity between a human liver carcinoma cell line, HepG2, and a clone of human embryonic kidney cells, TSA201. We defined the region between nucleotides −131 and −114, especially nucleotides −126 and −125, of the *hDIO1* promoter as critical for basal and LXRα-mediated specific transcriptional activation in HepG2 cells. An increase in *hDIO1* expression was observed in LXRα-stimulated cells, but absent in cycloheximide-treated cells, indicating that new protein synthesis is required for LXRα-mediated regulation of *hDIO1*. On the other hand, electrophoretic mobility shift assays revealed that LXRα and RXRα bound to the *hDIO1* promoter. We also demonstrated that LXRα and TRβ compete with each other on this specific region of the promoter. In conclusion, our results indicated that LXRα plays a specific and important role in activation of TH by regulating D1, and that LXRα binds to and regulates the *hDIO1* promoter, competing with TRβ on specific sequences within the promoter.

## Introduction

Iodothyronine deiodinases type 1, 2, and 3 (D1, D2, and D3, respectively) are selenoenzymes that regulate the activity of thyroid hormone (TH) via specific inner- or outer-ring deiodination [1]. Biologically active intracellular triiodothyronine (T3) is supplied from the serum and via activation of thyroxine (T4) by D1 and D2 [1]. In human, D1 is expressed at high levels in the liver, kidney, and thyroid, whereas D2 is expressed in the brain, pituitary, and brown adipose tissue. The local T3 concentration in hepatic cells depends on serum TH concentration, T3 generated from T4 by D1, and clearance of T3 from hepatic cells. The expression and activity of D1 are modulated by multiple factors including T3, cAMP, retinoic acid, and TSH [2–4]. In a previous report, we demonstrated that expression of the human D1 gene (*hDIO1*) is liver-specifically and cooperatively regulated by forkhead box A1 (FOXA1), FOXA2, and upstream stimulatory factor (USF) via specific regions of the *hDIO1* promoter [5]. However, much remains unknown regarding the mechanisms of regulation of *hDIO1* and its physiological role in humans.

Liver X receptors (LXRs) are ligand-activated transcription factors of the nuclear receptor superfamily that bind oxidized cholesterol and function as sensors for excess intracellular oxysterols [6]. LXRs exist in two isoforms, LXRα and LXRβ. LXRβ is expressed ubiquitously, whereas LXRα is highly expressed in the liver, and at lower levels in adipose tissue, adrenal glands, intestines, lungs, kidneys, and cells of myeloid origin [7]. LXRs form heterodimers with the retinoid X receptor (RXR) and bind to the LXR-responsive element (LXRE) in promoters of target genes that contain direct repeats (DRs) of the core sequence AGGTCA separated by four nucleotides (DR4) [8].

LXRs bind to and compete with another subgroup of nuclear receptor, the thyroid hormone receptors (TRs), at the same sites in the 5´-region of promoters, e.g., those of the genes encoding acetyl-CoA carboxylase-α, cholesterol 7-alpha-hydroxylase, and ATP-binding cassette transporter A1 [9–11]. Toyoda *et al*. previously reported that the *hDIO1* promoter contains two functional TH response elements (TREs), each of which also consists of two DRs with the core sequence, AGGTCA [3]. Although LXRs and TRs belong to two distinct receptor subgroups with respect to ligand-binding affinity [12], the two receptor systems have similar molecular mechanisms, i.e., both form heterodimers with RXRs and to bind to DR4 with identical geometry and polarity [13,14]. To date, it remains unknown whether LXRs regulate the expression of *hDIO1*. Because LXRα is expressed at very high levels in the liver, and the *hDIO1* promoter contains TREs, we hypothesized that LXRα is involved in regulation of *hDIO1* in a liver-specific manner by unknown mechanisms.

Thus, in this study, we investigated the involvement of LXRα in the regulation of *hDIO1*, in the liver using a human liver carcinoma cell line HepG2.

## Materials and methods

### Ligand

A synthetic LXR agonist, T0901317 (TO), was purchased from Cayman Chemical (Ann Arbor, MN, USA), and a synthetic LXR antagonist, GSK2033 (GSK), was purchased from Axon Medchem BV (Groningen, The Netherlands). TO and GSK were dissolved in and diluted with dimethyl sulfoxide at various concentrations.

### Cell culture

A human liver carcinoma cell line, HepG2 [5], and a clone of human embryonic kidney 293 cells, TSA201 [15], were cultured as described previously.

### Plasmid construction

Deletion mutants of the 5´-flanking region of *hDIO1* (−2023, −803, −187, −131, −113, −103/−4; the transcription start site was defined as +1, nucleotide 53894211, NC_000001.11) were prepared and subcloned into pGL 4.10 (Promega, Madison, WI, USA) to create a fusion with the luciferase gene (−2023, −803, −187, −131, −113, −103/−4 hDIO1-Luc) as described previously [5]. Mutations were created in the −131/−4 hDIO1-Luc construct at nucleotides −124, −119/−118, −108/−106, −126/−125, or both −126/−125 and −108/−106 of the *hDIO1* promoter using −131/−4 hDIO1-Luc construct as a template and the QuikChange® Site-Directed Mutagenesis kit (Stratagene, La Jolla, CA, USA). Sequences of the 5´-flanking region of mutated constructs are provided in Table 1. These constructs were verified by sequencing. Each plasmid expressing cDNA for human TRβ, human RXRα, human LXRα, and human farnesoid X receptor (FXR) was subcloned into pCMX as described previously [16]. A plasmid expressing cDNA for human pregnane X receptor (pFN21A-hPXR) was generated by Kazusa DNA Research Institute (Chiba, Japan) and purchased from Promega. We also prepared a plasmid expressing cDNA for human PAR-related orphan receptor alpha (RORα). The open reading frame of RORα was generated by PCR using cDNA from HepG2 cells as a template. We used primers containing *Sgf*I or *Pme*I linker: 5´-GACCGCGATCGCCATGGAGTCAGCTCCGGCAGCCC-3´ and 5´-ATTAGTTTAAACCCCATCAATTTGCATTGCATTGCTGGTCA-3´. The PCR product was digested with *Sgf*I and *Pme*I and cloned into *Sgf*I/*Pme*I-digested pFN21A CMV Flexi vector (Promega). The plasmid was verified by sequencing and synthesis of RORα was confirmed by western blot.

**Table 1:** Wild-type and mutated sequence of constructs used in mutational analysis.

Mutated construct and control	Sequence of 5´-flanking region of the construct between the nucleotides −131 and −104
−126/−125, −108/−106 Mut −131/−4 hDIO1-Luc	5´-TCTGA***AA***TGACTCCTTCCCCTGA***AAA***GG
−126/−125 Mut −131/−4 hDIO1-Luc	5´-TCTGA***AA***TGACTCCTTCCCCTGACCCGG
−124 Mut −131/−4 hDIO1-Luc	5´-TCTGACC***C***GACTCCTTCCCCTGACCCGG
−119/−118 Mut −131/−4 hDIO1-Luc	5´-TCTGACCTGACT***TG***TTCCCCTGACCCGG
−108/−106 Mut −131/−4 hDIO1-Luc	5´-TCTGAAATGACTCCTTCCCCTGA***AAA***GG
−131/−4 hDIO1-Luc	5´-TCTGACCTGACTCCTTCCCCTGACCCGG

The mutated base pairs between nucleotides −131 and −104 of the *hDIO1* promoter are indicated by bold italic letters with underlines.

### Transient transfection and luciferase assay

Transient transfections were performed in 24-well tissue culture plates using the Lipofectamine^TM^ LTX regent (Thermo Fisher Scientific, Waltham, MA, USA) for HepG2 cells, and the Lipofectamine^TM^ 2000 reagent (Thermo Fisher Scientific) for TSA201 cells, as described previously [5]. Briefly, cells were seeded in 24-well plates 1 day before transfection and maintained in 0.5 ml of antibiotic-free medium supplemented with 10% charcoal-stripped bovine serum (Thermo Fisher Scientific). For transfection of HepG2 cells, we used 500 ng of experimental reporter constructs, 50 ng of each expression vector, and 25 ng of pGL 4.74, which contains the cDNA encoding *Renilla* luciferase (Promega) as an internal control for transfection efficacy. For transfection of TSA201 cells, we used 100 ng of experimental reporter constructs, 10 ng of each expression vector, and 5 ng of pGL 4.74. In both cell types, culture media were replaced with antibiotic-free medium supplemented with 10% charcoal-stripped bovine serum with vehicle or 10^−7^ M TO 6–8 h after transfection. The cells were then cultured for an additional 48 h and harvested. Luciferase activity was determined using the Dual-Luciferase Reporter Assay System (Promega), and luminescence was measured by a 2030 ARVO*X* multilabel reader (PerkinElmer, Waltham, MA, USA). Firefly luciferase activity was normalized against *Renilla* luciferase activity in each well to control for transfection efficacy.

### RNA isolation, reverse transcription, and quantitative PCR

HepG2 cells were cultured in antibiotic-free medium supplemented with 10% charcoal-stripped bovine serum (Thermo Fisher Scientific) and treated with TO at various concentrations and 10^−6^ M GSK, or pre-treated with cycloheximide for 30 min before treatment with 10^−7^ M TO for 24 h. Then, total RNA was extracted from HepG2 cells using the RNeasy® Plus Mini Kit (QIAGEN, Valencia, CA, USA). One microgram of total RNA was reverse-transcribed with random hexamers using First-strand cDNA Synthesis Kit (GE Healthcare UK Ltd. Buckinghamshire, UK). The resultant cDNA was diluted 1:10 by addition of TE buffer.

Quantitative PCR was performed, recorded, and analyzed using TaqMan® Gene Expression Assays with the StepOnePlus™ Real-time PCR System (Thermo Fisher Scientific). The probe/primer sets were Hs00174944_m1 (*hDIO1*), Hs00230861_m1 (*THRB*), and Human *PPIA* (Cyclophilin A) Endogenous Control, purchased from Thermo Fisher Scientific. Diluted cDNA was amplified as described previously [5]. Expression levels of each gene were normalized against the corresponding mRNA levels of cyclophilin A to compensate for variations in input RNA.

### Preparation of electrophoresis mobility shift assay (EMSA)

Nuclear extracts were prepared from HepG2 cells and TSA201 cells using the Nuclear Extract Kit (Active Motif, Carlsbad, CA, USA). HepG2 cells were treated with vehicle or TO, with or without co-transfection of expression vectors for LXRα and RXRα (LXRα/ RXRα). One microgram of each expression vector was transfected into HepG2 cells in 60-mm dishes using the Lipofectamine^TM^ LTX regent (Thermo Fisher Scientific). Vehicle or TO was added to the culture media 24 h after transfection. The cells were then cultured for an additional 24 h and harvested. EMSAs and supershift assays were conducted using the LightShift^TM^ Chemiluminescent EMSA kit (Thermo Fisher Scientific) as directed by the manufacturer with slight modifications, as described previously [5]. The antibodies used in the supershift assays were as follows: anti–human LXRα mouse monoclonal antibody (PP-PPZ0412-00, Perseus Proteomics, Tokyo, Japan), anti–human RXRα mouse monoclonal antibody (PP-K8508-00, Perseus Proteomics), anti–human TRβ1 mouse monoclonal antibody (sc-738X, Santa Cruz Biotechnology, Dallas, TX, USA), and normal mouse IgG (sc-2025, Santa Cruz Biotechnology). The sequences of oligonucleotides used for EMSA are provided in Table 2.

**Table 2:** Sequences of double-stranded oligonucleotides used in EMSA.

Oligonucleotides	Sequences
Wt1	5´-GCAAACATCT**TCTGACCTGACTCCTTCC**CC-3´
−124 mut1	5´-GCAAACATCT**TCTGACC*****C*****GACTCCTTCC**CC-3´
−126/−125 mut1	5´-GCAAACATCT**TCTGA*****AA*****TGACTCCTTCC**CC-3´
Wt2	5´-**TCTGACCTGACTCCTT**CCCCTGACCCGG-3´
−126/−125 mut2	5´-**TCTGA*****AA*****TGACTCCTT**CCCCTGACCCGG-3´
LXRE	5´-GATCTTAGTTCACTCAAGTTCAAGGATC-3´

Wt1, oligonucleotide containing the wild-type sequence of the region between nucleotides −141 and −112 of the *hDIO1* promoter; −124 mut1, oligonucleotide containing a mutation at nucleotide −124 of the Wt1 oligonucleotide; −126/−125 mut1, oligonucleotide containing mutations at nucleotides −126 and −125 of the Wt1 oligonucleotide; Wt2, oligonucleotide containing the sequence of the region between nucleotides −131 and −104 of the *hDIO1* promoter [3]; −126/−125 mut2, oligonucleotide containing mutations at nucleotides −126 and −125 of the Wt2 oligonucleotide; LXRE, oligonucleotide containing the consensus sequence of the LXR response element [17]. The oligonucleotide sequence between nucleotides −131 and −114 of the *hDIO1* promoter is indicated by bold letters, and mutated base pairs are indicated by bold italic letters with underlines.

### Transfection of short interfering RNA (siRNA)

An aliquot of 2.5 pmol siRNA specific for *THRB* (s14121, Silencer Select^®^ siRNA, Thermo Fisher Scientific) or a negative control siRNA (Negative Control #2, Silencer Select^®^ siRNA, Thermo Fisher Scientific) was introduced into HepG2 cells using 24-well plates and the Lipofectamine RNAiMAX reagent (Thermo Fisher Scientific) by reverse transfection. Transfections of siRNA were performed 24 h before transfections of expression vectors for the luciferase assay. To determine the knockdown efficacy of siRNA, mRNA was extracted 72 h after siRNA transfection and analyzed by quantitative PCR as described above. Changes in levels of TRβ protein (coded by *THRB*) following siRNA transfections were verified by western blot. See S1 File for detailed information.

### Statistical analysis

Data are expressed as means ± standard error of the mean (SEM) obtained from at least three separate experiments. Significance of differences was evaluated using analysis of variance (ANOVA) followed by the Tukey–Kramer method, unless otherwise specified. *P* values < 0.05 were considered to be statistically significant.

## Results

### Identification of the specific region of the *hDIO1* promoter required for its basal activity and LXRα-mediated in HepG2 cells

**Fig 1:**
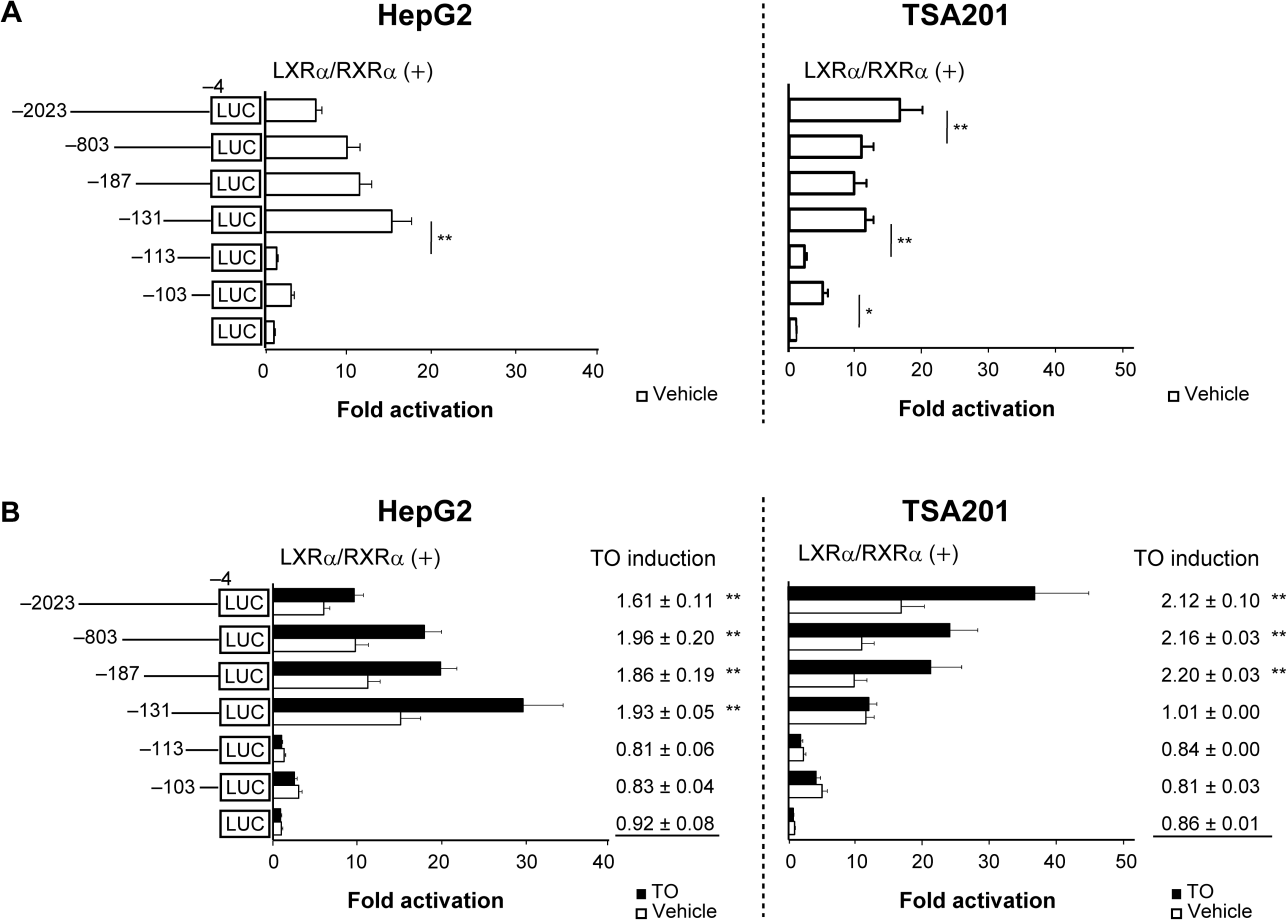
HepG2-specific regulation of the *hDIO1* promoter by T0901317 (TO). A series of 5´-deletion constructs of the *hDIO1* promoter were transiently transfected into HepG2 and TSA201 cells along with expression vectors for human LXRα and human RXRα (LXRα/RXRα) with and without 10^−7^ M TO. Promoter activity was normalized against *Renilla* luciferase activity, and the normalized value is expressed relative to that of promoterless pGL 4.10 in the absence of TO. Results are expressed as means ± SEM. *, *P* < 0.05; **, *P* < 0.01. A. Basal luciferase activity of each construct. Statistical analysis was performed on pairwise comparisons of constructs, and significant pairs are presented. B. Luciferase activities of each construct with and without 10^−7^ M TO. TO induction indicates ratio of promoter activity with TO to the activity without TO. Statistical analysis was performed to compare TO induction of each construct with that of promoterless pGL 4.10, and significant differences are presented.

To identify the specific region of the *hDIO1* promoter important for its activation by the synthetic LXR agonist, TO, we transiently transfected a series of 5´-deletion constructs into HepG2 and TSA201 cells, along with or without expression vectors for LXRα and RXRα (LXRα/RXRα). The cells were then cultured with or without TO (Fig 1 and S2 File). In both cell lines, basal luciferase activity was significantly decreased by deletion of nucleotides −131 to −114 (*P* < 0.01) (Fig 1A and Fig A in S2 File). Luciferase activity following addition of TO was significantly increased (1.48-fold) without expression vectors for LXRα/RXRα (Fig B in S2 File") and further increased (1.93-fold) with those vectors in HepG2 cells transfected with the −131/−4 hDIO1-Luc construct (*P* < 0.01) (Fig 1B). These increases in response to TO were abolished by deletion of nucleotides −131 to −114. On the other hand, in TSA201 cells transfected with the −131/−4 hDIO1-Luc construct, no significant increase in luciferase activity was observed in response to TO (Fig 1B). These results indicated the importance of the specific region between nucleotides −131 and −114 for both basal activity and LXRα**-**mediated activation of the *hDIO1* promoter in HepG2 cells.

### Regulation of *hDIO1* expression by LXRα

**Fig 2:**
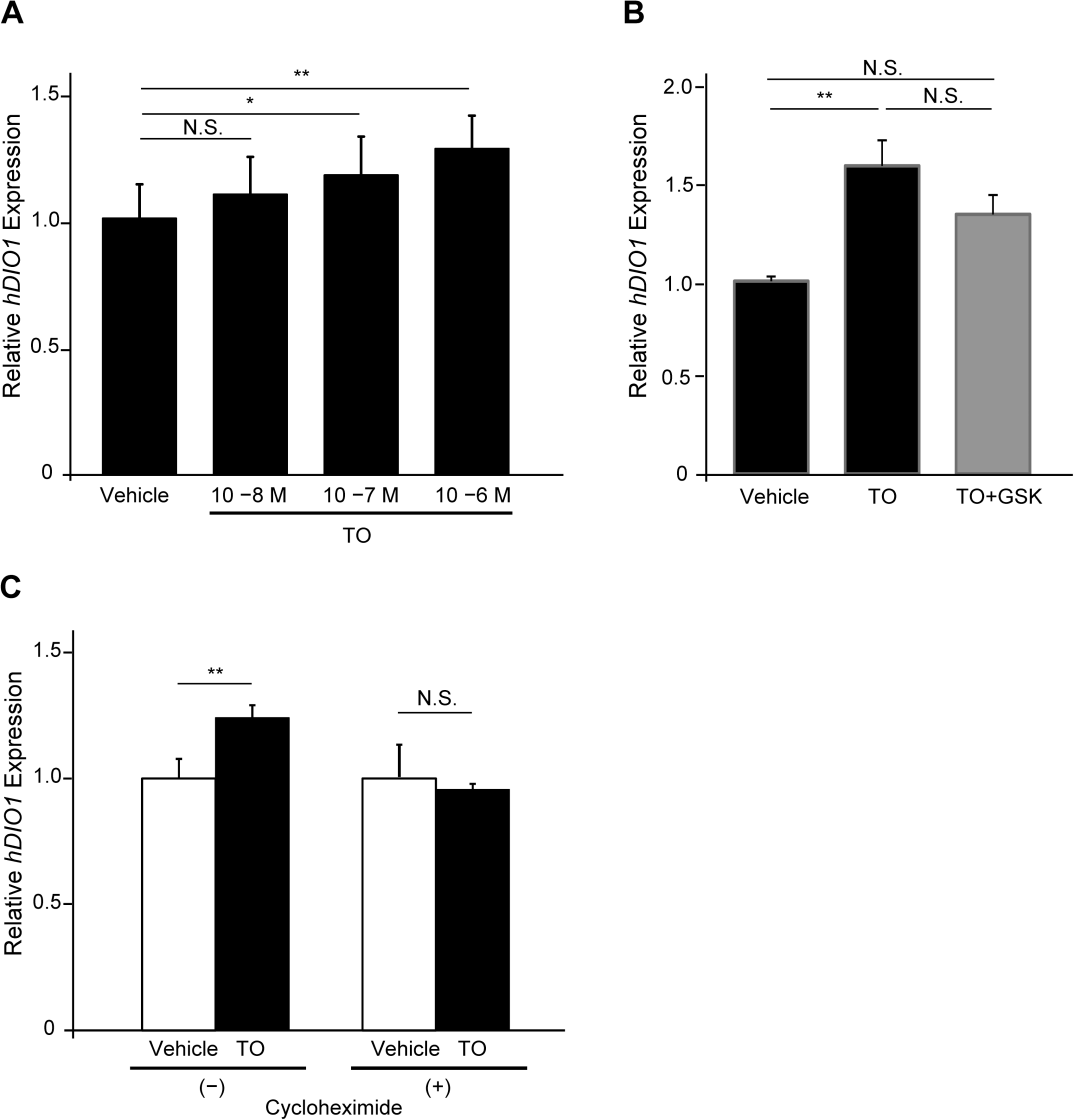
Analysis of relative *hDIO1* mRNA levels in HepG2 cells. *hDIO1* mRNA levels were normalized against the corresponding levels of cyclophilin A mRNA, and the value in HepG2/vehicle cells was defined as 1. Values are expressed as means ± SEM. *, *P* < 0.05; **, *P* < 0.01; N.S., not significant. A. HepG2 cells were treated at different concentrations of TO for 24 h. B. HepG2 cells were treated with vehicle, 10^−6^ M TO, or 10^−6^ M TO and 10^−6^ M GSK2033 (GSK) for 24 h. C. HepG2 cells were exposed to cycloheximide for 30 min before being treated with TO (10^−7^ M) for 24 h.

To confirm the change in *hDIO1* mRNA levels, we performed quantitative PCR analysis. The *hDIO1* mRNA level was increased by TO in a dose-dependent manner (Fig 2A). Furthermore, the increase in *hDIO1* mRNA following addition of TO was diminished by co-treatment with a LXR antagonist GSK (Fig 2B).

TO is a ligand for FXR, PXR, and RORα as well as LXRα [18,19]. To verify that the effect of TO on the *hDIO1* promoter depends on LXRα, we performed luciferase assays using expression vectors for these receptors in HepG2 cells (S3 File). Exclusively in cells co-transfected with expression vectors for LXRα/RXRα, luciferase activity in response to TO was significantly higher than in cells transfected with empty vectors. Overexpression of FXR and RORα did not alter luciferase activity, and PXR instead decreased, in comparison with the results of each empty vector. These results indicated that LXRα specifically activates the *hDIO1* promoter.

To determine whether LXRα needs *de novo* protein synthesis to stimulate *hDIO1* transcription, we performed the TO stimulation experiment using HepG2 cells pre-treated with cycloheximide. In this case, the mRNA level of *hDIO1* was not increased by TO (Fig 2C).

### Investigation of the key nucleotides within the region between nucleotides −131 and −114 of the *hDIO1* promoter

**Fig 3:**
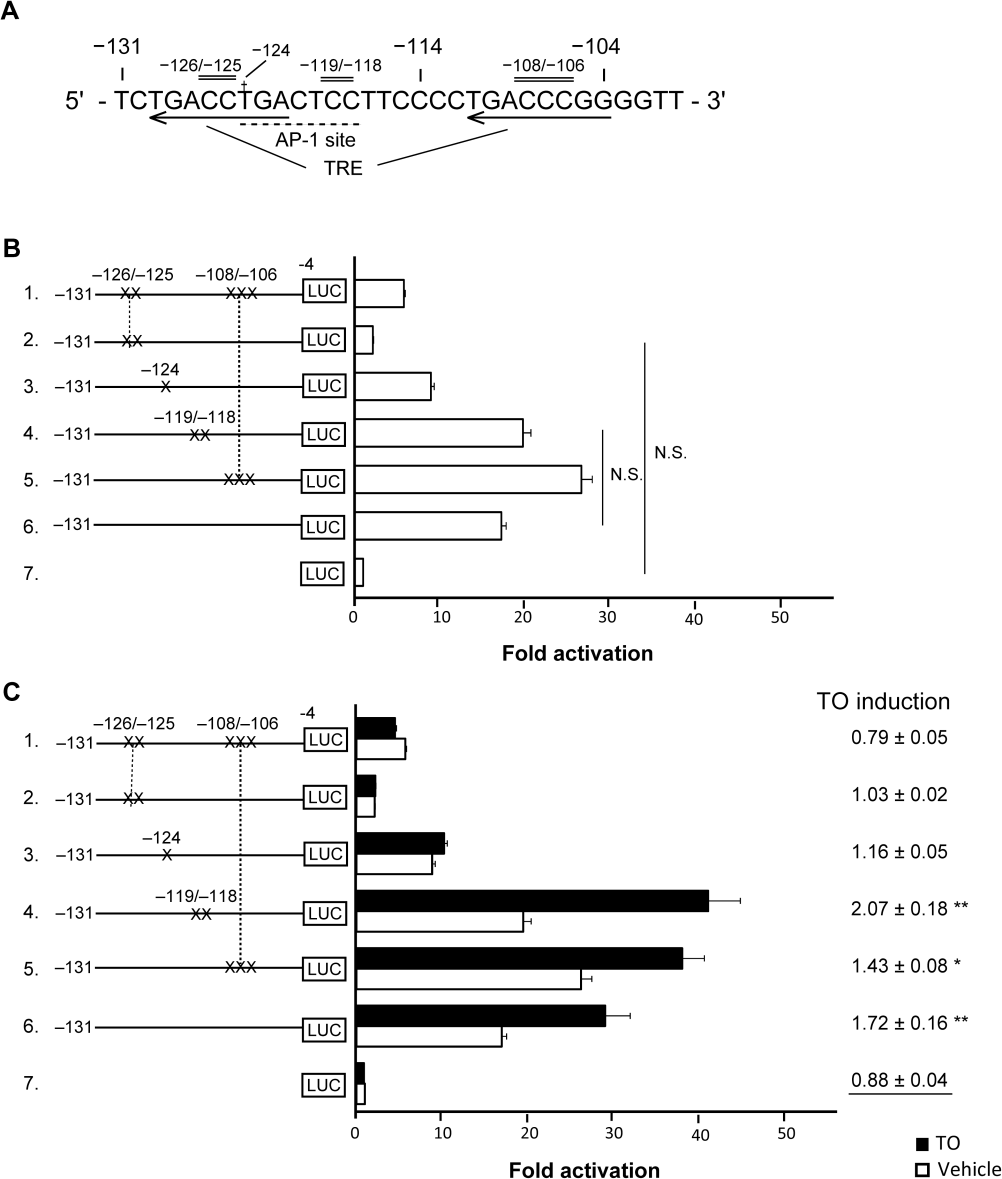
Mutational analysis of activation of the *hDIO1* promoter by T0901317 (TO). A. The nucleotide sequence of the 5´-flanking region of the *hDIO1* promoter used in the analysis, along with the positional relationship among mutated oligonucleotides, thyroid hormone response element (TRE), and the putative activator protein 1 (AP-1) site. † and black horizontal bars represent the site-specific mutation. B and C. A series of mutated *hDIO1* promoter constructs was transiently transfected into HepG2 cells along with expression vectors for LXRα/RXRα, with or without 10^−7^ M TO. Schematic diagram to the left of the figure representing mutant (No. 1–5) and wild-type (No. 6) *hDIO1* promoters, which were introduced upstream of the luciferase gene. No. 7 represents the promoterless pGL 4.10 construct. Promoter activity was normalized against *Renilla* luciferase activity, and is expressed relative to that of promoterless pGL 4.10 in the absence of TO. Values are expressed as means ± SEM. *, *P* < 0.05; **, *P* < 0.01; N.S., not significant. B. Basal luciferase activity of each construct. Statistical analysis was performed on comparisons between all constructs. Because most pairs exhibited significance, only non-significant pairs are presented. C. Luciferase activities of each construct with and without 10^−7^ M TO. TO induction indicates ratios of promoter activity with TO to the activity without TO. Statistical analysis was performed on comparisons of TO induction of each construct with that of promoterless pGL 4.10, and significant differences are presented.

To narrow down the specific site required for the basal activity and LXRα-mediated activation of the *hDIO1* promoter, we performed luciferase assays using HepG2 cells transfected with wild-type or mutant *hDIO1* promoter–reporter constructs. From the previous reports of Toyoda et al. [3] and a computational analysis [20], the region between nucleotides −131 and −114 of the *hDIO1* promoter contains a half-site of TRE, whose binding site consists of two octamer half-site motifs (YYRGGTCA) separated by 10 bp [3], as well as an activator protein 1 (AP-1) site, whose consensus sequence contains TGA(C/G) TCA [21–23], as shown in Fig 3A. Thus, mutations were introduced to disrupt the sequences constituting the TRE [3] and putative AP-1 site (Fig 3A). Basal luciferase activity was significantly reduced when nucleotides −126/−125 or nucleotide −124 was mutated, in comparison to the construct lacking any mutations (Fig 3B); in the former case, the reduction was larger, reaching the baseline level (i.e., the level in cells transfected with promoterless pGL 4.10). On the other hand, basal luciferase activity increased when nucleotides −108/−106 were mutated, but did not change when nucleotides −119/−118 were mutated (Fig 3B). The increase in luciferase activity of the *hDIO1* promoter by TO was abolished when nucleotides −126/−125 or a nucleotide −124 was mutated, but not when nucleotides −108/−106 or −119/−118 were mutated (Fig 3C).

### Identification of nuclear proteins that bind to nucleotides −131 to −114 of the *hDIO1* promoter in HepG2 and TSA201 cells

**Fig 4:**
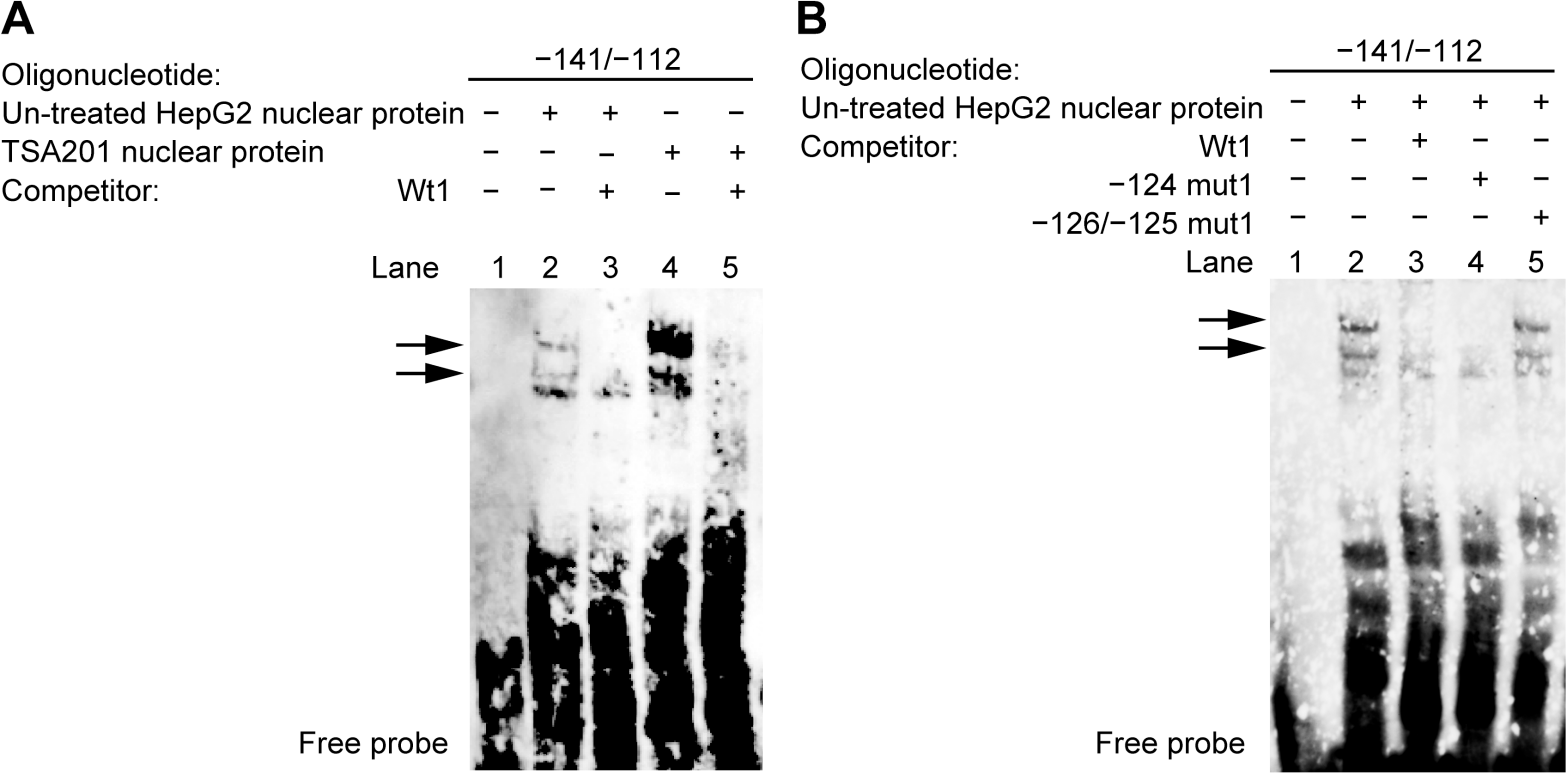
Specific binding of transcription factors to the region between nucleotides −131 and −114 of the *hDIO1* promoter. A. EMSA with oligonucleotide containing the wild-type sequence of the region between nucleotides −141 and −112 of the *hDIO1* promoter (Wt1) and nuclear extracts from un-treated HepG2 cells and TSA201 cells. In lane 1, as a control, only the biotin-labeled Wt1 oligonucleotide was present. Biotin-labeled Wt1 oligonucleotide was incubated with nuclear extracts from HepG2 or TSA201 cells without competitors in lanes 2 and 4, respectively, and with 25-fold molar excesses of unlabeled Wt1 oligonucleotides in lanes 3 and 5, respectively. The specific DNA/protein complexes formed are indicated by arrows. B. EMSA with mutant oligonucleotides and nuclear extracts from HepG2 cells. Biotin-labeled Wt1 oligonucleotide was incubated with nuclear extracts from HepG2 cells without competitors in lane 2, and with 25-fold molar excesses of unlabeled Wt1, −124mut1, and −126/−125mut1 oligonucleotides in lanes 3, 4, and 5, respectively. The specific DNA/protein complexes formed are indicated by arrows.

To confirm the interaction between DNA and proteins within nucleotides −131 to −114 of the *hDIO1* promoter, we performed EMSA using oligonucleotides containing the wild-type sequence of the region between nucleotides −141 and −112 of the *hDIO1* promoter (Wt1) (Fig 4A). Incubation of nuclear proteins from HepG2 cells or TSA201 cells with biotin-labeled Wt1 oligonucleotides led to the formation of several DNA/protein complexes, as shown in lanes 2 and 4, respectively. In both cell lines, specific formation of these complexes was inhibited by incubation with excess unlabeled Wt1 oligonucleotides (lanes 3 and 5).

Furthermore, we performed EMSA using nuclear extracts from HepG2 cells and mutated unlabeled nucleotides (−124 mut1 or −126/−125 mut1) (Fig 4B). The same DNA/protein complexes observed in previous experiments (lane 2) were abolished when the nuclear proteins were incubated with excess unlabeled Wt1 oligonucleotides (lane 3), but not with excess unlabeled −126/−125 mut1 oligonucleotides (lane 5), supporting the idea that the specific DNA/protein complexes formed within the region between nucleotides −131 and −114 of the *hDIO1* promoter. However, both DNA/protein complexes were abolished when nuclear extracts were incubated with excess unlabeled −124 mut1 oligonucleotides (lane 4).

### Binding of LXRα and RXRα on the *hDIO1* promoter

**Fig 5:**
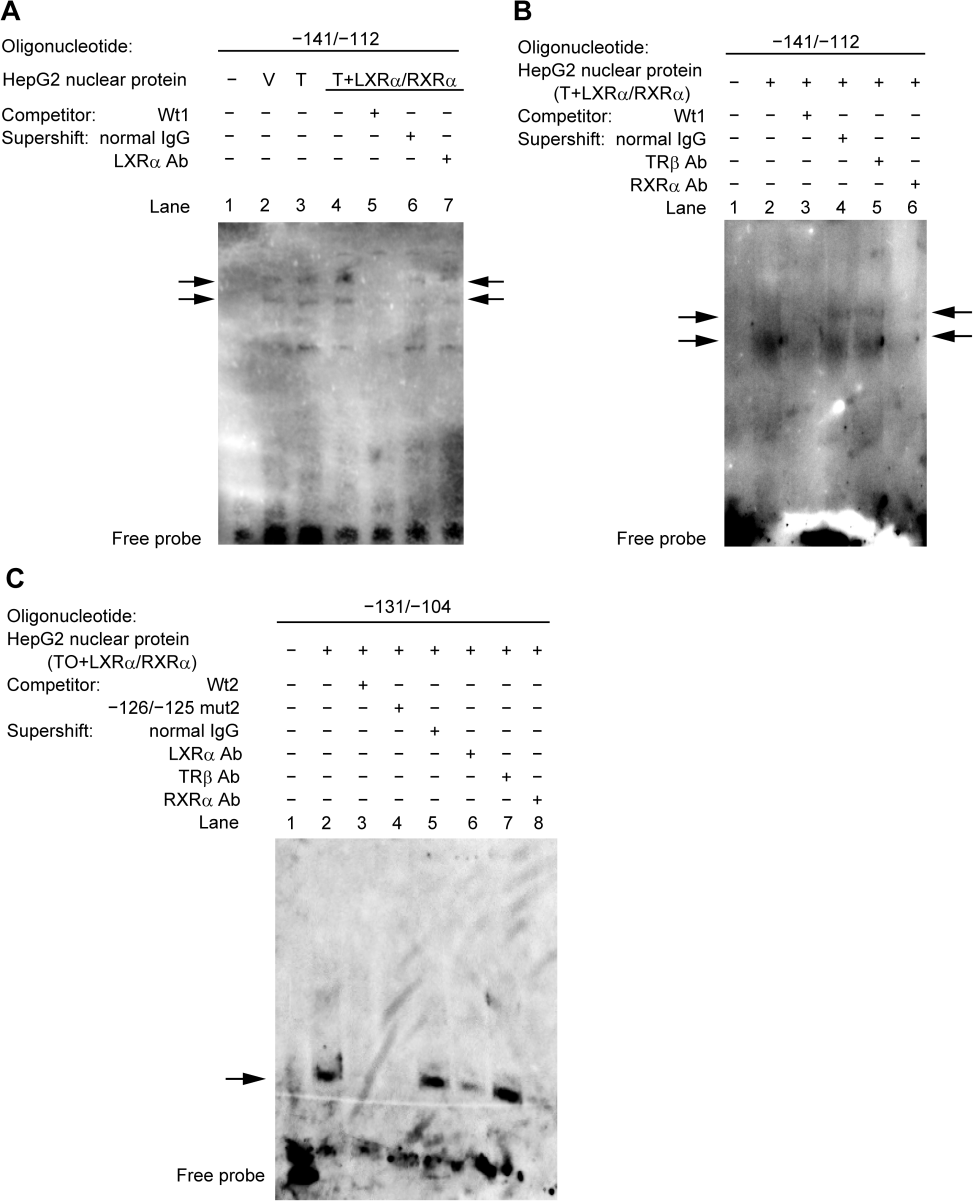
Binding of LXRα on the *hDIO1* promoter. A. EMSA with oligonucleotide containing the wild-type sequence of the region between nucleotides −141 and −112 of the *hDIO1* promoter (Wt1) with nuclear extracts from vehicle-treated (V), T0901317 (TO)-treated (T), or TO-treated and LXRα/RXRα-overexpressing (T+LXRα/RXRα) HepG2 cells; also shown is a supershift assay with an antibody against LXRα. Specific DNA/protein complexes are indicated by arrows. In lane 1, as a control, only biotin-labeled Wt1 oligonucleotide was present. In lanes 2, 3, and 4, biotin-labeled Wt1 oligonucleotide was incubated with nuclear extracts from V, T, and T+LXRα/RXRα HepG2 cells without competitor, respectively. In lane 5, biotin-labeled Wt1 oligonucleotide was incubated with nuclear extracts from T+LXRα/RXRα HepG2 cells with excess unlabeled Wt1 oligonucleotides as competitor. The results of the supershift assay are shown with normal mouse IgG as a control in lane 6 and with an antibody against LXRα in lane 7. B. EMSA with Wt1 oligonucleotides with nuclear extracts from T+LXRα/RXRα HepG2 cells and a supershift assay with antibodies against TRβ and RXRα. Specific DNA/protein complexes are indicated by arrows. In lane 1, as a control, only biotin-labeled Wt1 oligonucleotide was present. Biotin-labeled Wt1 oligonucleotide was incubated with the nuclear extracts in lane 2 and with the nuclear extracts and excess unlabeled Wt1 oligonucleotides as competitor in lane 3. The results of the supershift assay are shown with normal mouse IgG as a control in lane 4, with an antibody against TRβ in lane 5, and with an antibody against RXRα in lane 6. C. EMSA was performed with oligonucleotides containing the wild-type sequence of the region between nucleotides −131 and −104 of the *hDIO1* promoter (Wt2) with nuclear extracts from T+LXRα/RXRα HepG2 cells; also shown is a supershift assay with antibodies against LXRα, TRβ, and RXRα. A specific DNA/protein complex is indicated by an arrow. In lane 1, as a control, only biotin-labeled Wt2 was present. Biotin-labeled Wt2 was incubated with nuclear extracts in lane 2, with nuclear extracts and excess unlabeled Wt2 oligonucleotides as competitor in lane 3, and with excess unlabeled −126/−125 mut2 as competitor in lane 4. The results of the supershift assay are shown with normal mouse IgG as a control in lane 5, with an antibody against LXRα in lane 6, with an antibody against TRβ in lane 7, and with an antibody against RXRα in lane 8.

The results of EMSA using nuclear extracts from vehicle-treated, TO-treated, and TO-treated and LXRα/RXRα-overexpressing HepG2 cells were shown in Fig 5A. The DNA/protein complexes were observed at the same positions and appeared slightly stronger using nuclear extracts from TO-treated cells (lane 3) and much stronger using nuclear extracts from TO-treated and LXRα/RXRα-overexpressing cells (lane 4), in comparison with those using nuclear extracts from vehicle-treated cells (lane 2).

To identify the protein that binds this region, we performed supershift assays using antibodies against LXRα, TRβ, and RXRα with nuclear extracts from TO-treated and LXRα/RXRα-overexpressing HepG2 cells (Fig 5A and 5B). The results of validation of the antibodies used in supershift assays are shown in S4 File. The supershift of the complexes was not observed with antibodies against LXRα (Fig 5A, lane 7) or TRβ (Fig 5B, lane 5), but was observed with an antibody against RXRα (Fig 5B, lane 6). These results indicated that the DNA/protein complexes formed within nucleotides −131 to −114 of the *hDIO1* promoter contained RXRα.

Considering that RXRα forms a heterodimer with other nuclear receptors on the 3´ side of RXRα binding position, such as LXRα and TRβ [24], and that nucleotides −131 to −114 contain a half-site of TRE while nucleotides −113 to −104 contain another half-site, we performed EMSA using an oligonucleotide containing the sequence of the region between nucleotides −131 and −104 (Wt2) [3] with nuclear extracts from TO-treated and LXRα/RXRα-overexpressing HepG2 cells (Fig 5C). As a result, a DNA/protein complex was formed (lane 2), but this complex abolished when the nuclear extracts were incubated with excess unlabeled Wt2 oligonucleotides (lane 3) or excess unlabeled −126/−125 mut2 oligonucleotides (lane 4). In a supershift assay, the formed complex was supershifted with antibodies against both LXRα and RXRα (lanes 6 and 8), but not TRβ (lane 7). These results indicated that LXRα, as well as RXRα, can bind to the region between nucleotides −131 and −104 of the *hDIO1* promoter. In addition, unlike RXRα binding, LXRα binding also required the region between nucleotides −113 and −104.

Furthermore, to determine whether LXRα binds to the *hDIO1* promoter under physiological conditions, we performed ChIP assays using an antibody against LXRα. We described materials and methods in S1 File. Using both primer sets, approximately 6-fold to 8-fold enrichment of ChIP-DNA following LXRα immunoprecipitation was observed in comparison with the negative control (S5 File), indicating that LXRα could physiologically bind to the *hDIO1* promoter.

### Interaction of LXRα/RXRα and TRβ on a specific region of the *hDIO1* promoter

**Fig 6:**
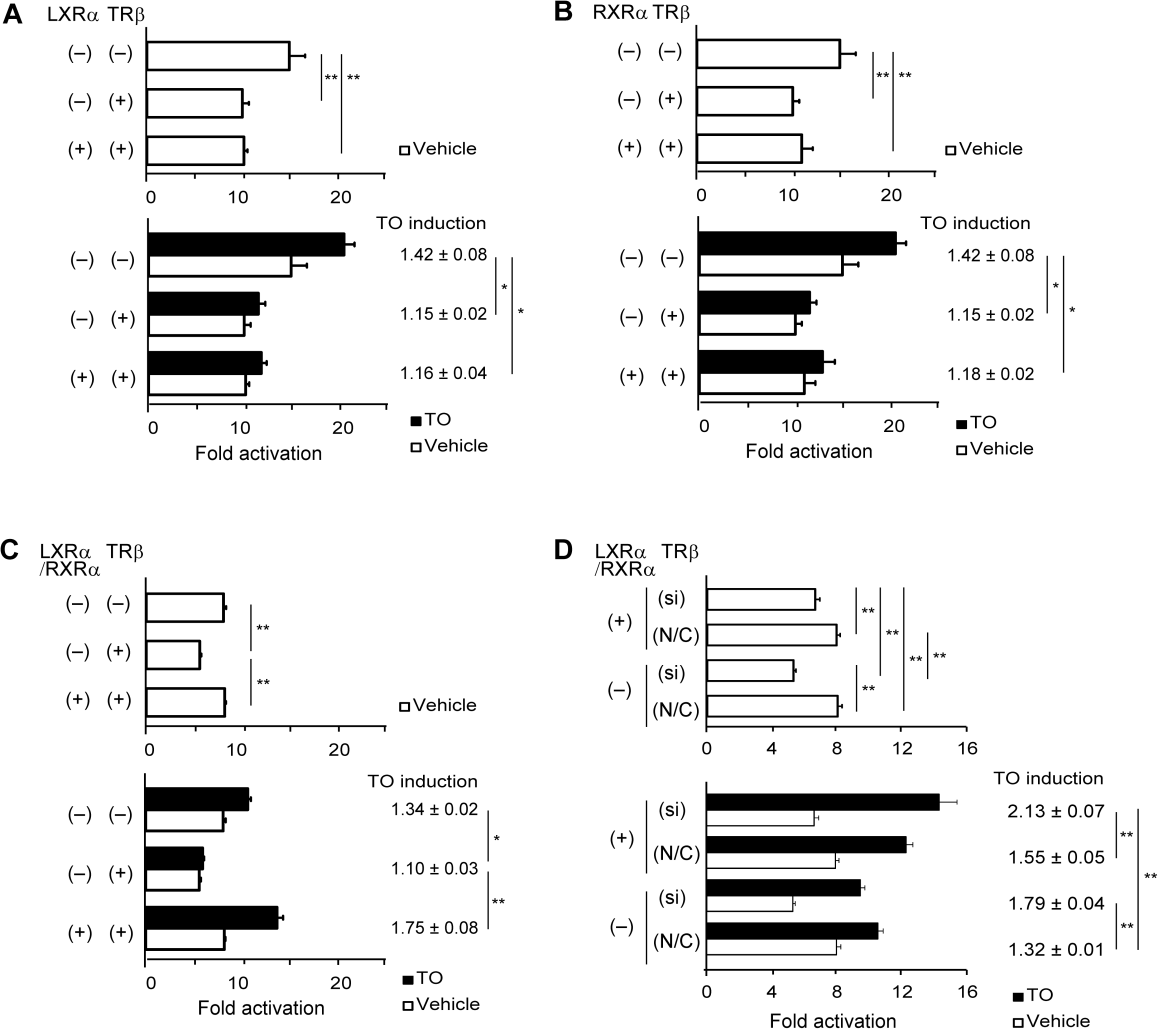
Interaction of LXRα/RXRα and TRβ on the activity of the *hDIO1* promoter. HepG2 cells were transfected with or without expression vectors for LXRα, RXRα, or LXRα/RXRα, with or without TRβ or siRNA specific for *THRB*. We performed luciferase assay using a −131/−4 hDIO1-Luc construct. All upper figures show only basal luciferase activities, whereas lower figures show both basal activities and activities with or without 10^−7^ M TO. TO induction indicates ratios of promoter activity with TO to the activity without TO. Promoter activity was normalized against *Renilla* luciferase activity, and the normalized value is expressed relative to that of promoterless pGL 4.10 in the absence of TO. Results are expressed as means ± SEM. Statistical analysis was performed on comparisons among all conditions; the results of basal activity are shown in the upper figure, and those of TO induction are shown in the lower figure; *, *P* < 0.05; **, *P* < 0.01. A. Comparison with and without overexpression of LXRα or TRβ. B. Comparison with and without overexpression of RXRα or TRβ. C. Comparison with and without overexpression of LXRα/RXRα or TRβ. D. Comparison with and without overexpression of LXRα/RXRα and TRβ knockdown using siRNA. si, siRNA specific for *THRB*; N/C, negative control siRNA.

TRs bind to or compete with LXRs on the same regions in the promoters of some genes [9–11], and basal suppression of gene promoters by un-liganded TRs has been reported [25,26]. Therefore, we examined the interaction of LXRα, RXRα, and TRβ on a specific region of the *hDIO1* promoter using the −131/−4 hDIO1-Luc construct (Fig 6). Basal luciferase activity, which was significantly decreased by overexpression of TRβ, was not abolished when expression vector for either LXRα or RXRα was transfected alone (Fig 6A and 6B), but was abolished when expression vectors for LXRα/RXRα were co-transfected (Fig 6C). Similarly, overexpression of TRβ decreased TO induction, except when expression vectors for LXRα/RXRα were co-transfected (Fig 6C).

Next, we examined TO induction in TRβ-knockdown conditions (Fig 6D). The siRNA specific for TRβ efficiently knocked down the *THRB* mRNA and the encoded protein (S6 File). Basal luciferase activity was significantly increased in TRβ-knockdown cells co-transfected with expression vectors for LXRα/RXRα. Furthermore, in TRβ-knockdown cells, TO induction was increased (from 1.32-fold to 1.79-fold) in the absence of co-transfection of expression vectors for LXRα/RXRα, and further increased (from 1.55-fold to 2.13-fold) in the presence of LXRα/RXRα. These results indicated that LXRα/RXRα compete with TRβ in TO induction, as well as basal activity, in this specific region of the *hDIO1* promoter.

## Discussion

In this study, we investigated the involvement and mechanism of regulation of *hDIO1* mediated by LXRα using the synthetic LXR agonist TO. We identified a specific region between nucleotides −131 and −114 of the *hDIO1* promoter that was important for basal activity and LXRα-mediated activation of *hDIO1* specifically in HepG2 cells. The results of mutational analysis showed that basal and TO-induced luciferase activities of the *hDIO1* promoter were abolished when nucleotides −126/−125 or a nucleotide −124 was mutated. EMSA using nuclear extracts from HepG2 and TSA201 cells revealed that some DNA/protein complexes were formed on oligonucleotides containing the sequence of the region between nucleotides −131 and −114 of the *hDIO1* promoter. Supershifts of DNA/protein complexes were observed with an antibody against RXRα using oligonucleotides containing the sequence of the region between nucleotides −131 and −114, as well as with antibodies against LXRα and RXRα using the oligonucleotides containing the sequence of the region between nucleotides −131 and −104. In addition, luciferase assays revealed that LXRα/RXRα and TRβ compete with each other on this specific region of the *hDIO1* promoter when TRβ is either overexpressed or knocked down.

The specific region we identified between nucleotides −131 and −114 of the *hDIO1* promoter contains a TRE, as reported by Toyoda et al. [3]. Within this region, nucleotides −126/−125 were critical for both basal and LXRα-mediated activation and protein binding on the *hDIO1* promoter. This result is consistent with reports that the CC residues within TRE are the most important determinants for TRβ binding [27,28]. Luciferase assays revealed that basal and LXRα-mediated activation was also abolished when a nucleotide −124 was mutated. However, based on the results of EMSA using excess mutated unlabeled oligonucleotide, a nucleotide −124 (unlike nucleotides −126/−125) did not seem to be essential for formation of DNA/protein complexes.

We used a synthetic LXR ligand, TO, in this study. Some synthetic ligands for LXRs, such as TO and GW3965, were developed because oxysterols, the natural ligands for LXRs, have relatively low affinities for LXRs [29,30]. We selected TO as a ligand for LXRs for our *in vitro* study because it is a stronger activator of LXRs than GW3965 in HepG2 cells [31]. TO is a potential activator of other nuclear receptors including FXR, PXR, and RORα [18,19]. To confirm that TO specifically stimulates LXRα to regulate *hDIO1*, we used two strategies. First, we confirmed that the activation of luciferase activity in response to TO was not augmented by overexpression of FXR, PXR, or RORα, but was augmented by LXRα/RXRα. Second, the response to TO in *hDIO1* mRNA was antagonized by a LXR antagonist GSK.

The results of EMSA and supershift assay suggested that DNA/protein complexes, including LXRα and RXRα were formed with oligonucleotides containing the sequences of the region between the nucleotides −131 and −104 of the *hDIO1* promoter. The supershift with an antibody against RXRα was observed not only with an oligonucleotide containing the sequences of the region between nucleotides −141 and −112 of the *hDIO1* promoter (containing the sequences of the 5´ single half-site of TRE), but also with an oligonucleotide containing the sequences of the region between nucleotides −131 and −104 (containing the sequences of complete TRE). On the other hand, the supershift with an antibody against LXRα was observed only with the oligonucleotide containing the sequences of the region between nucleotides −131 and −104. These results suggest that LXRα has effects on the region between nucleotides −111 and −104 of the *hDIO1* promoter, where the 3´ half-site of TRE is located.

RXR forms heterodimers with numerous other nuclear receptors, including retinoic acid receptor, vitamin D receptor, TRs, and LXRs [8,32,33]. The consensus sequence of RXR, 5´-AGGTCA-3´ [34], is located between nucleotides −129 and −124 of the *hDIO1* promoter, supporting our EMSA results showing that supershift with an antibody against RXRα was observed with oligonucleotides containing the sequences of the regions between nucleotides −131 and −114 and nucleotides −131 and −104. In addition, interactions mediated by dimerization of RXRα with other receptors occur only when RXR binds to the 5´ half-site of their binding positions [35]. The importance of the polarity of RXRα was consistent with the results of the supershift assay with an antibody against LXRα. We observed that the DNA/protein complexes were more prominent when nuclear extracts from TO-treated (and especially TO-treated and LXRα/RXRα-overexpressing) HepG2 cells were used. This suggests that the DNA/protein complexes might contain some other proteins induced by TO or overexpression of LXRα/RXRα. Cycloheximide treatment abolished the increase in mRNA levels of *hDIO1* by TO, suggesting that some newly synthesized proteins are involved in stimulation of *hDIO1* transcription by LXRα.

Interactions between TR and LXR have been reported on promoters of some genes [9–11,36]. We confirmed that TR and LXR interact on the *hDIO1* promoter; TO induction was significantly decreased by overexpression of TRβ, but this effect was abolished by co-transfection of expression vectors for LXRα/RXRα. Interestingly, the change in TO induction by TRβ was not affected by transfection of expression vector for either LXRα or RXRα alone. This highlights the relevance of the EMSA/supershift results showing that complexes including both LXRα and RXRα bound to the specific region between nucleotides −131 and −104 of the *hDIO1* promoter. By contrast, in TRβ-knockdown cells, TO induction was significantly increased with or without overexpression of LXRα/RXRα, indicating that deterioration of TRβ function augments the effects of LXRα/RXRα on the *hDIO1* promoter.

Our observation that stimulation of LXRα by TO increased the expression of *hDIO1* in HepG2 cells was inconsistent with previous *in vivo* observations that TO decreases hepatic expression of *Dio1* in rat and mouse models [37,38]. Importantly, there is a large difference in the sequences of this gene’s promoter regions between human and rodent; TREs have not been identified in the promoter regions of rat or mouse *Dio1*, whereas two TREs are present in the promoter region of human *DIO1* [3]. Therefore, we performed *in vitro* examinations using human cell lines, and obtained meaningful data on human *DIO1* gene.

In conclusion, we demonstrated that a specific region of the *hDIO1* promoter, which contains a TRE, was important for the basal activity and LXRα-mediated activation of this gene. Our results provide the additional insights that LXRα plays a specific and important role in activation of TH by regulating D1. Furthermore, LXRα binds to and regulates the *hDIO1* promoter, and competes with TRβ on a specific region of the *hDIO1* promoter.

## Supporting information

S1 File Supplemental materials and methods. (DOCX)

S2 File A series of 5'-deletion analysis of the *hDIO1* promoter without LXRα/RXRα overexpression. A. A series of 5'-deletion constructs of the *hDIO1* promoter were transiently transfected into HepG2 and TSA201 cells in the absence of expression vectors for human LXRα and human RXRα (LXRα/RXRα), with or without 10^−7^ M TO. Promoter activity was normalized against *Renilla* luciferase activity, and the normalized value is expressed relative to that of promoterless pGL 4.10 in the absence of TO. Results are expressed as means ± SEM. **, P < 0.01. A. Basal luciferase activity of each construct. Statistical analysis was performed for pairwise combinations of constructs, and significant pairs are presented. B. Luciferase activities of each construct with and without 10^−7^ M TO. TO induction indicates ratios of promoter activity with TO vs. without TO. Statistical analysis was performed between TO induction of each construct and that of promoterless pGL 4.10, and significant differences are presented.
(TIF)

S3 File Specificity of T0901317 (TO) in the regulation of *hDIO1* by LXRα. HepG2 cells were treated with and without 10^−7^ M TO and transfected using expression vectors as follows. pCMX-LXRα, co-transfection with pCMX-LXRα and pCMX-RXRα; pCMX-FXR, co-transfection with pCMX-FXR and pCMX-RXRα; pCMX-empty, transfection of pCMX-empty vector alone; pFN21A-RORα, co-transfection of pFN21A-RORα and pCMX-empty vector; pFN21A-PXR, co-transfection of pFN21A-PXR and pCMX-RXRα; and pFN21A-empty, co-transfection of pCMX-empty vector and pFN21A-empty vector. Promoter activity was normalized against *Renilla* luciferase activity, and the normalized value is expressed relative to that of promoterless pGL 4.10 with pCMX-empty or pFN21A-empty in the absence of TO. TO induction indicates ratios of promoter activity with TO vs. without TO. Statistical analysis was performed to compare each group with the respective empty vector group. Results are expressed as means ± SEM. *, P < 0.05; **, P < 0.01; N.S., not significant.
(TIF)

S4 File Positive control for antibodies against TRβ, RXRα, and LXRα. A. EMSA with oligonucleotide containing the wild-type sequence of the region between nucleotides −131 and −104 of the *hDIO1* promoter (Wt2) with nuclear extracts from HepG2 cells overexpressing TRβ and RXRα, and supershift assays with antibodies against TRβ and RXRα. Specific DNA/protein complexes are indicated by black arrows and supershifted bands are by a gray arrow. In lane 1, as a control, only biotin-labeled Wt2 oligonucleotide was present. Biotin-labeled Wt2 oligonucleotide was incubated with nuclear extracts in lane 2 and with nuclear extracts and excess unlabeled Wt2 oligonucleotides as competitor in lane 3. The results of supershift assays are shown with normal mouse IgG as a control in lane 4, with an antibody against TRβ in lane 5, and with an antibody against RXRα in lane 6. B. EMSA with oligonucleotide containing consensus sequence of LXR response element (LXRE) with nuclear extracts from HepG2 cells overexpressing LXRα and RXRα, and supershift assays with an antibody against LXRα. Specific DNA/protein complexes are indicated by black arrows and a supershifted band is by a gray arrow. In lane 1, as a control, only biotin-labeled LXRE oligonucleotide was present. Biotin-labeled LXRE oligonucleotide was incubated with nuclear extracts in lane 2 and with nuclear extracts and excess unlabeled LXRE oligonucleotides as competitor in lane 3. The results of supershift assays are shown with normal mouse IgG as a control in lane 4 and with an antibody against LXRα in lane 5.
(TIF)

S5 File Binding of LXRα on the *hDIO1* promoter. We purified chromatin fragments following immunoprecipitation from HepG2 cells using an antibody against LXRα. Normal mouse IgG was used as a negative control for an antibody against LXRα. The presence of specific DNA fragments binding to LXRα was determined by quantitative PCR using primer set 1 and 2, which covers nucleotides −128/+41 and −188/−25 of the genomic *hDIO1* promoter, respectively. Enrichment of LXRα-bound DNA and that of normal mouse IgG-bound DNA were normalized against 10% of the corresponding input DNA, and the results of enrichment of LXRα-bound DNA were expressed as the fold change relative to normal mouse IgG-bound DNA. Data were compared by unpaired Student’s t test. Values are expressed as means ± SEM. **, P < 0.01.
(TIF)

S6 File Efficiency of siRNA-mediated knockdown of TRβ. A. The results of quantitative PCR to detect *THRB* mRNA. We compared HepG2 cells transfected with negative control siRNA (N/C) or siRNA against *THRB* (si-TRβ). *THRB* mRNA levels were normalized against the corresponding levels of cyclophilin A mRNA, and the value in N/C was defined as 1. Values are expressed as means ± SEM. **, P < 0.01. B. Protein levels of TRβ after siRNA transfection were evaluated by western blot analysis. β-actin was used as a standard.
(TIF)
