# Supplementary material for: Regulation of type 1 iodothyronine deiodinase by LXRα
Source: PLoS One. 2017 Jun 15;12(6):e0179213. doi: 10.1371/journal.pone.0179213 (PMC5472309; doi:10.1371/journal.pone.0179213)
Supplement: S1 File — (DOCX) [file pone.0179213.s001.docx]

**S1 File. Supplemental materials and methods.**

**Chromatin immunoprecipitation (ChIP) assay**

ChIP assays were performed using the ChIP-IT Express kit (Active Motif, Carlsbad, CA, USA) according to the manufacturer’s instructions, with the following modifications to improve efficiency. HepG2 cells were cultured in 15-cm culture dishes to 70% confluence, and the media were replaced with 20 ml of antibiotic-free medium supplemented with 10% charcoal-stripped bovine serum (Thermo Fisher Scientific, Waltham, MA, USA). The cells were then cultured for 24 h and harvested. Crosslinked nuclear pellets were sonicated for 10 min on a Covaris S220 (Covaris, MA, USA). Chromatin fragments were incubated overnight at 4 °C with specific antibodies and protein G magnetic beads on an end-to-end rotator. Antibodies were anti-human LXRα　mouse monoclonal antibody (PP-PPZ0412-00, Perseus Proteomics, Tokyo, Japan) and normal mouse IgG (sc-2025, Santa Cruz Biotechnology, Dallas, TX, USA), used as a negative control. Subsequently, the beads were washed with ChIP buffer 1 and ChIP buffer 2, followed by resuspension of the beads in 100 µl of elution buffer (10 mM Tris-HCl pH 8.0, 1 mM EDTA, 0.5% SDS) and elution at 65 °C for 30 min in a thermal cycler. The eluates were incubated with 10 ng/µl of RNase A for 30 min at 37 °C, and then reverse-crosslinked with 0.4 mg/ml of proteinase K for 6 h at 65 °C in a thermal cycler. DNA was purified using Mini Elute PCR Purification Kit (QIAGEN, Valencia, CA, USA). The samples were analyzed by quantitative PCR using THUNDERBIRD^®^ SYBR qPCR Mix (Toyobo, Osaka, Japan) and LightCycler^®^ 480 (Roche Diagnostics, Mannheim, Germany). The resultant signals were normalized against the corresponding levels of 10% input DNA for each primer pair. The primers for quantitative PCR used in this study are listed in Table 3.

**Table 3. Sequences of primers used in ChIP assay**

| Primer set | Primer sequences (5'–3') | | Region covering genomic sequences |
| --- | --- | --- | --- |
|  |  |  | / Genomic coordinates |
| 1 | Forward | GACCTGACTCCTTCCCCTGA | −128/+41 genomic *hDIO1* |
|  | Reverse | CAGAGCCTCTTCAGCCACAG | / nucleotide 53894083 to 53894251, NC_000001.11 |
| 2 | Forward | AGACCTTTGTGCACCTGGTT | −188/−25 genomic *hDIO1* |
|  | Reverse | GAGTTCTATGGGCAGAGAGGC | / nucleotide 53894023 to 53894186, NC_000001.11 |

Transcription start site of genomic *hDIO1* was defined as +1.

**Western blot**

We electrophoresed 16 μg/lane of nuclear extracts in Bolt 4–12% Bis-Tris Plus Gels (Thermo Fisher Scientific) and transferred the protein samples onto polyvinylidene difluoride membranes with iBlot Dry Blotting System (Thermo Fisher Scientific).　The membranes were blocked with Blocking One (Nacalai Tesque, Kyoto, Japan), incubated with primary antibody overnight at 4 °C, and then incubated with secondary antibody for 2 h at room temperature. All antibodies were diluted in Can Get Signal Immunoreaction Enhancer Solution (TOYOBO, Osaka, Japan). Primary antibodies were mouse monoclonal antibody against TRβ1 (sc-738X, Santa Cruz Biotechnology) at 1:500 dilution and rabbit polyclonal antibody against β-actin (4967; Cell Signaling Technology, Danvers, MA, USA) at 1:2500 dilution. The secondary antibodies used were rabbit anti–mouse IgG-HRP (sc-358914, Santa Cruz Biotechnology) and donkey anti–rabbit IgG-HRP (sc-2077, Santa Cruz Biotechnology). We detected bands by a chemiluminescent method using Chemi-Lumi One Super (Nacalai Tesque) on an ImageQuant LAS 4000 (GE Healthcare, Chicago, IL, USA).
