# Supplementary figures and images for: Regulation of type 1 iodothyronine deiodinase by LXRα
Source: PLoS One. 2017 Jun 15;12(6):e0179213. doi: 10.1371/journal.pone.0179213 (PMC5472309; doi:10.1371/journal.pone.0179213)

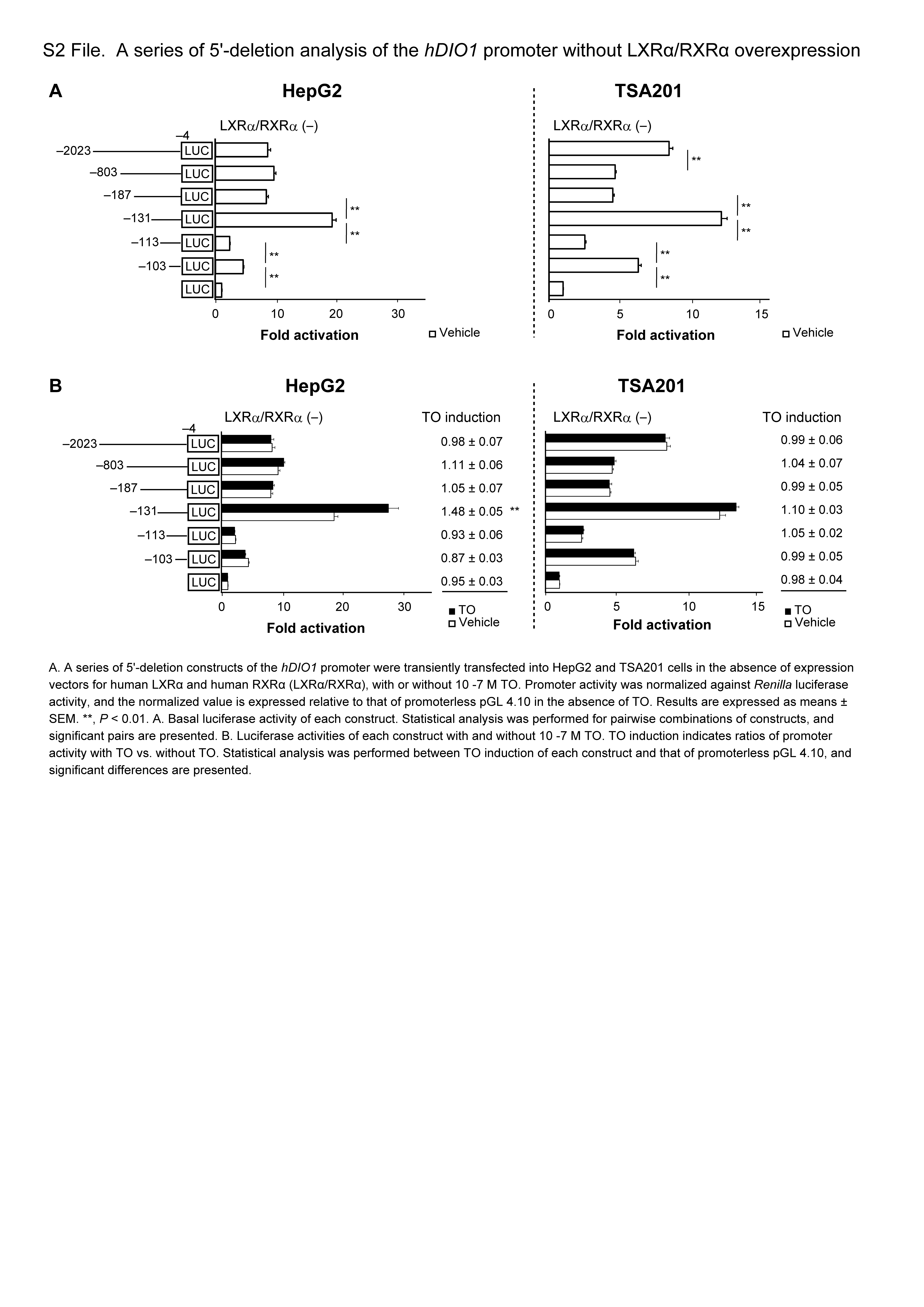

Supplement: S2 File — A. A series of 5'-deletion constructs of the hDIO1 promoter were transiently transfected into HepG2 and TSA201 cells in the absence of expression vectors for human LXRα and human RXRα (LXRα/RXRα), with or without 10−7 M TO. Promoter activity was normalized against Renilla luciferase activity, and the normalized value is expressed relative to that of promoterless pGL 4.10 in the absence of TO. Results are expressed as means ± SEM. **, P < 0.01. A. Basal luciferase activity of each construct. Statistical analysis was performed for pairwise combinations of constructs, and significant pairs are presented. B. Luciferase activities of each construct with and without 10−7 M TO. TO induction indicates ratios of promoter activity with TO vs. without TO. Statistical analysis was performed between TO induction of each construct and that of promoterless pGL 4.10, and significant differences are presented. (TIF) [file pone.0179213.s002.tif]

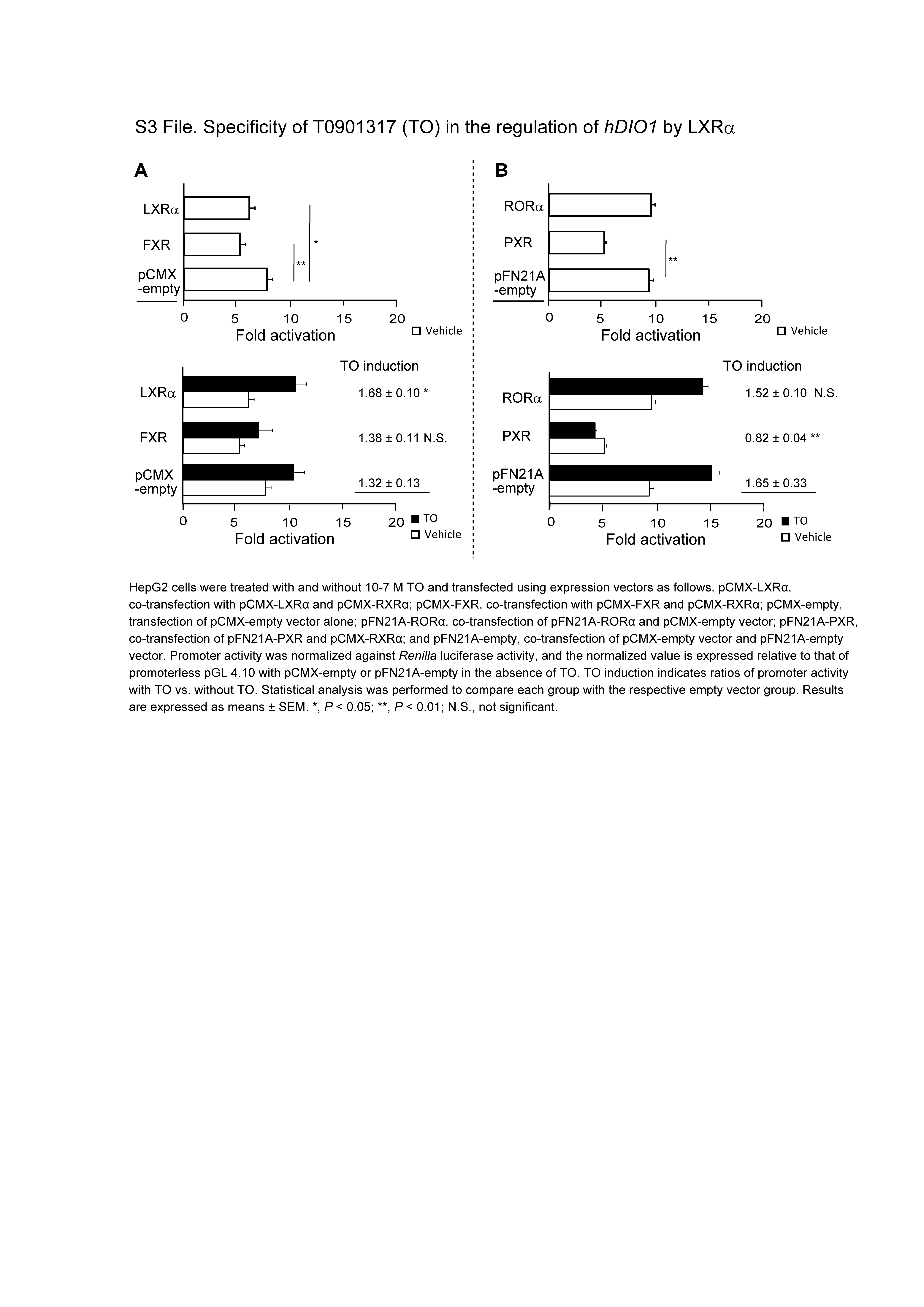

Supplement: S3 File — HepG2 cells were treated with and without 10−7 M TO and transfected using expression vectors as follows. pCMX-LXRα, co-transfection with pCMX-LXRα and pCMX-RXRα; pCMX-FXR, co-transfection with pCMX-FXR and pCMX-RXRα; pCMX-empty, transfection of pCMX-empty vector alone; pFN21A-RORα, co-transfection of pFN21A-RORα and pCMX-empty vector; pFN21A-PXR, co-transfection of pFN21A-PXR and pCMX-RXRα; and pFN21A-empty, co-transfection of pCMX-empty vector and pFN21A-empty vector. Promoter activity was normalized against Renilla luciferase activity, and the normalized value is expressed relative to that of promoterless pGL 4.10 with pCMX-empty or pFN21A-empty in the absence of TO. TO induction indicates ratios of promoter activity with TO vs. without TO. Statistical analysis was performed to compare each group with the respective empty vector group. Results are expressed as means ± SEM. *, P < 0.05; **, P < 0.01; N.S., not significant. (TIF) [file pone.0179213.s003.tif]

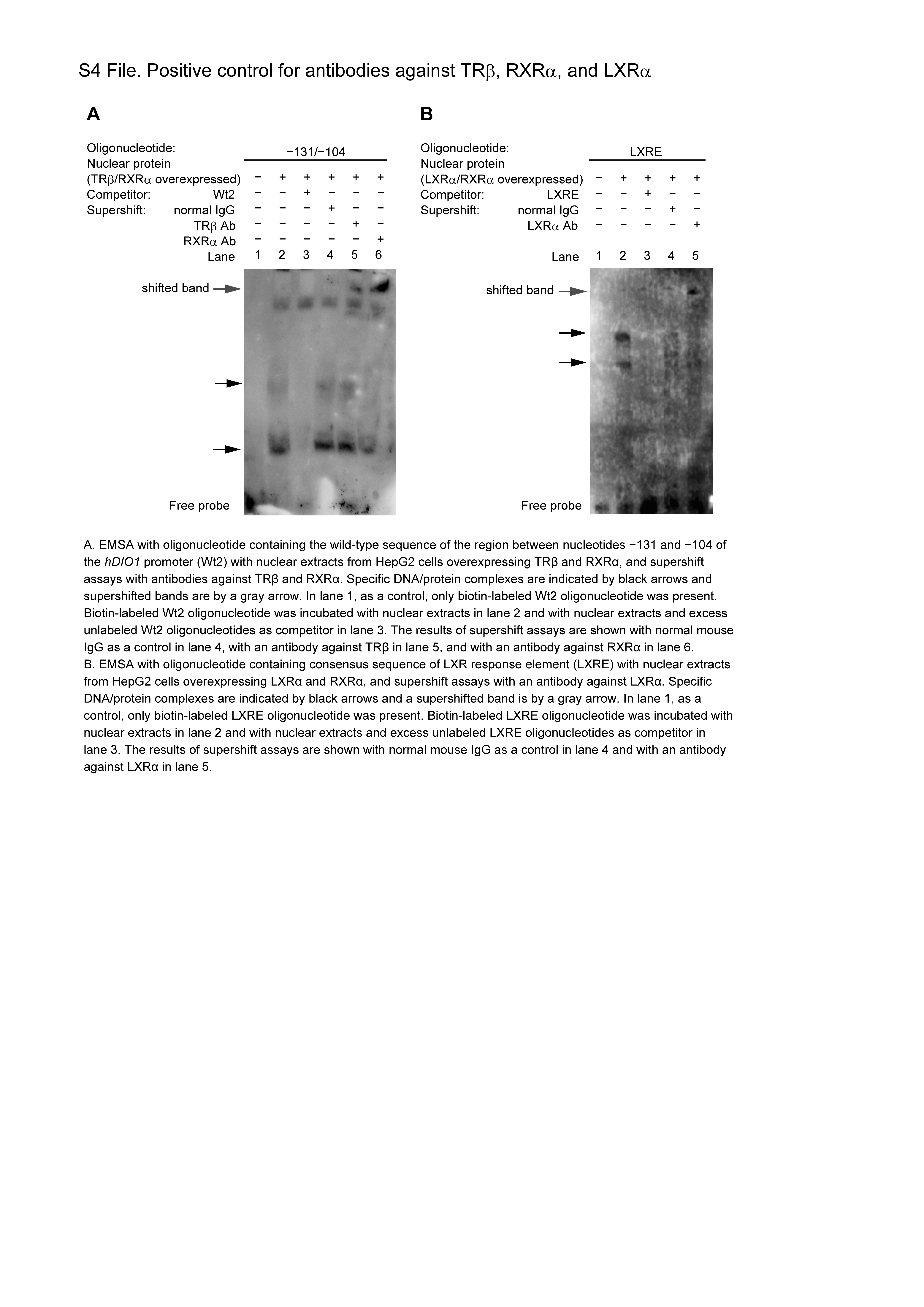

Supplement: S4 File — A. EMSA with oligonucleotide containing the wild-type sequence of the region between nucleotides −131 and −104 of the hDIO1 promoter (Wt2) with nuclear extracts from HepG2 cells overexpressing TRβ and RXRα, and supershift assays with antibodies against TRβ and RXRα. Specific DNA/protein complexes are indicated by black arrows and supershifted bands are by a gray arrow. In lane 1, as a control, only biotin-labeled Wt2 oligonucleotide was present. Biotin-labeled Wt2 oligonucleotide was incubated with nuclear extracts in lane 2 and with nuclear extracts and excess unlabeled Wt2 oligonucleotides as competitor in lane 3. The results of supershift assays are shown with normal mouse IgG as a control in lane 4, with an antibody against TRβ in lane 5, and with an antibody against RXRα in lane 6. B. EMSA with oligonucleotide containing consensus sequence of LXR response element (LXRE) with nuclear extracts from HepG2 cells overexpressing LXRα and RXRα, and supershift assays with an antibody against LXRα. Specific DNA/protein complexes are indicated by black arrows and a supershifted band is by a gray arrow. In lane 1, as a control, only biotin-labeled LXRE oligonucleotide was present. Biotin-labeled LXRE oligonucleotide was incubated with nuclear extracts in lane 2 and with nuclear extracts and excess unlabeled LXRE oligonucleotides as competitor in lane 3. The results of supershift assays are shown with normal mouse IgG as a control in lane 4 and with an antibody against LXRα in lane 5. (TIF) [file pone.0179213.s004.tif]

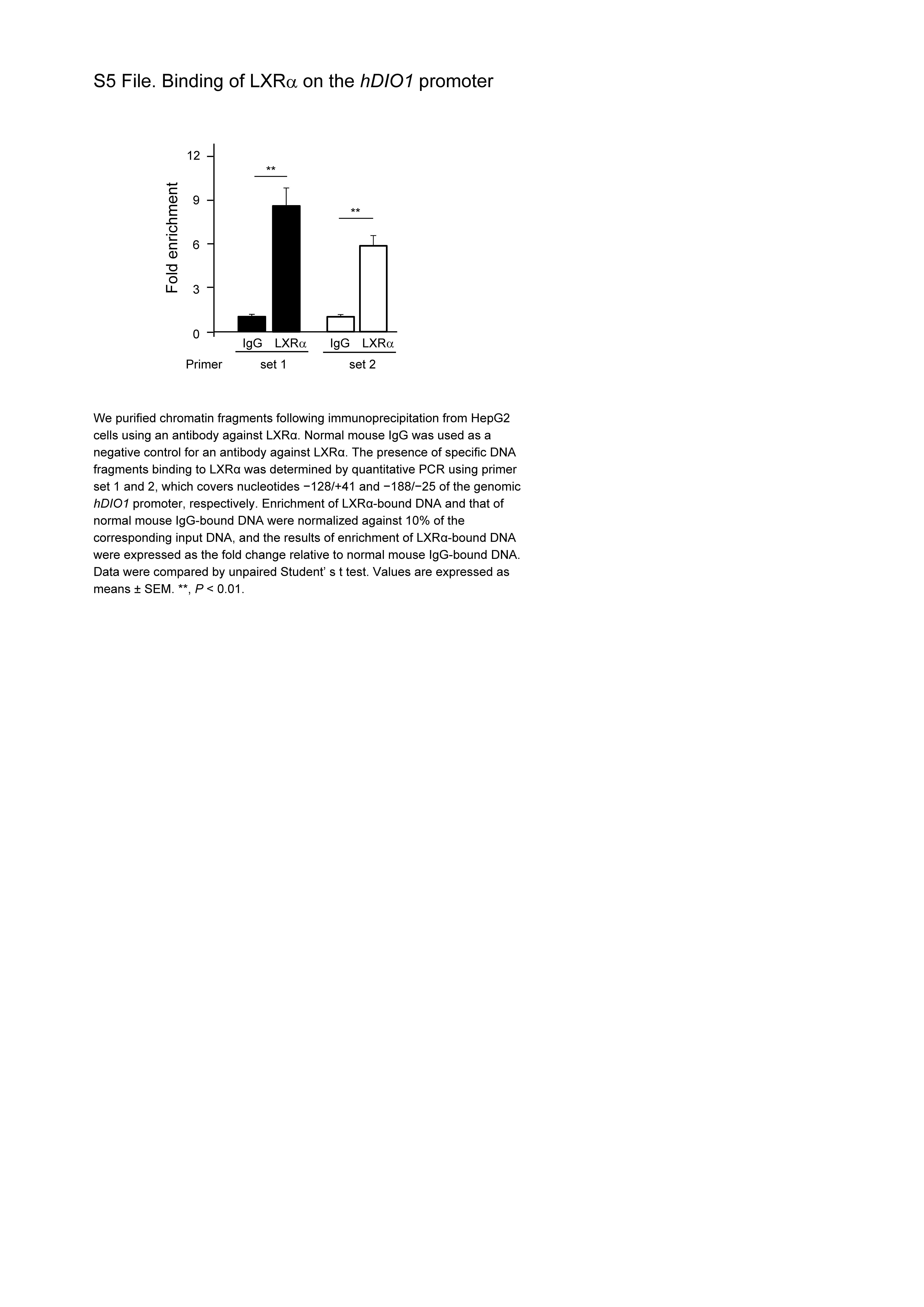

Supplement: S5 File — We purified chromatin fragments following immunoprecipitation from HepG2 cells using an antibody against LXRα. Normal mouse IgG was used as a negative control for an antibody against LXRα. The presence of specific DNA fragments binding to LXRα was determined by quantitative PCR using primer set 1 and 2, which covers nucleotides −128/+41 and −188/−25 of the genomic hDIO1 promoter, respectively. Enrichment of LXRα-bound DNA and that of normal mouse IgG-bound DNA were normalized against 10% of the corresponding input DNA, and the results of enrichment of LXRα-bound DNA were expressed as the fold change relative to normal mouse IgG-bound DNA. Data were compared by unpaired Student’s t test. Values are expressed as means ± SEM. **, P < 0.01. (TIF) [file pone.0179213.s005.tif]

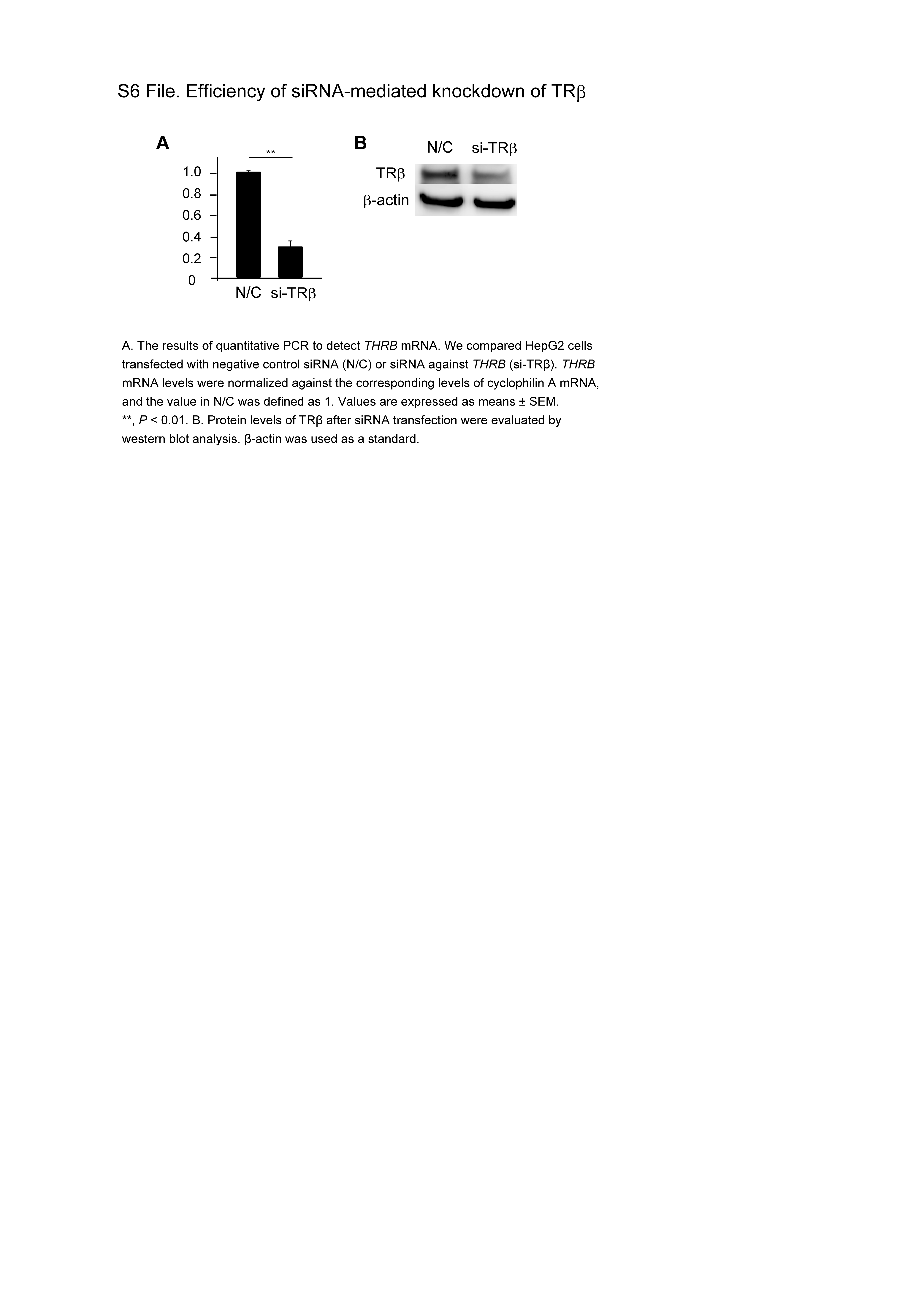

Supplement: S6 File — A. The results of quantitative PCR to detect THRB mRNA. We compared HepG2 cells transfected with negative control siRNA (N/C) or siRNA against THRB (si-TRβ). THRB mRNA levels were normalized against the corresponding levels of cyclophilin A mRNA, and the value in N/C was defined as 1. Values are expressed as means ± SEM. **, P < 0.01. B. Protein levels of TRβ after siRNA transfection were evaluated by western blot analysis. β-actin was used as a standard. (TIF) [file pone.0179213.s006.tif]
